# Current Concepts in Multi-Modality Imaging of Solid Tumor Angiogenesis

**DOI:** 10.3390/cancers12113239

**Published:** 2020-11-03

**Authors:** Moataz A. Soliman, Jeffrey Guccione, Anna M. Reiter, Ahmed W. Moawad, Ashley Etchison, Serageldin Kamel, Aline D. Khatchikian, Khaled M. Elsayes

**Affiliations:** 1 Department of Diagnostic Radiology, Northwestern University, Evanston, IL 60201, USA; moataz.a.soliman@students.kasralainy.edu.eg; 2 Department of Diagnostic and Interventional Imaging, The University of Texas Health Sciences Center at Houston, Houston, TX 77030, USA; jeffrey.Guccione@uth.tmc.edu; 3 School of Medicine, University of Texas Southwestern, Dallas, TX 75390, USA; anna.Reiter@UTSouthwestern.edu; 4 Department of Diagnostic Radiology, The University of Texas M. D. Anderson Cancer Center, Houston, TX 77030, USA; ahmedw.moawad@mercyhealth.org; 5 Department of Diagnostic Radiology, Baylor College of Medicine, Houston, TX 76798, USA; ashley.Etchison@bcm.edu; 6 Department of Lymphoma and Myeloma, The University of Texas M. D. Anderson Cancer Center, Houston, TX 77030, USA; skattia@mdanderson.org; 7 Department of Diagnostic Radiology, McGill University, Montreal, QC H3G 1A4, Canada; aline.d.khatchikian@mail.mcgill.ca

**Keywords:** cancer imaging, anti-angiogenic therapy, treatment response, angiogenesis

## Abstract

**Simple Summary:**

The recent increase in the use of targeted molecular therapy including anti-angiogenetic agents in cancer treatment necessitate the use of robust tools to assess and guide treatment. Angiogenesis, the formation of new disorganized blood vessels, is used by tumor cells to grow and spread using different mechanisms that could be targeted by anti-angiogenetic agents. In this review, we discuss the biological principles of tumor angiogenesis and the imaging modalities that could provide information beyond gross tumor size and morphology to capture the efficacy of anti-angiogenetic therapeutic response.

**Abstract:**

There have been rapid advancements in cancer treatment in recent years, including targeted molecular therapy and the emergence of anti-angiogenic agents, which necessitate the need to quickly and accurately assess treatment response. The ideal tool is robust and non-invasive so that the treatment can be rapidly adjusted or discontinued based on efficacy. Since targeted therapies primarily affect tumor angiogenesis, morphological assessment based on tumor size alone may be insufficient, and other imaging modalities and features may be more helpful in assessing response. This review aims to discuss the biological principles of tumor angiogenesis and the multi-modality imaging evaluation of anti-angiogenic therapeutic responses.

## 1. Introduction

In the early 1970s, researchers first proposed that tumor growth is limited by an adequate blood supply. This concept propagated investigative research to explore various angiogenic factors, including the discovery of the human vascular endothelial growth factor (VEGF) in the 1980s. Eventually, researchers translated their growing knowledge about angiogenesis to clinical medicine by introducing angiogenesis inhibitors as a therapy for angiogenic diseases [1,2]. Due to the unique relationship between VEGF and malignant neovascularization, VEGF became the principal target of interest for anti-angiogenic drugs for cancer therapies. Introduced in 2004, bevacizumab, a human monoclonal antibody to VEGF, was the first such agent to receive FDA clearance. Initially, this drug was specifically approved for the treatment of colorectal cancer but has since received FDA approval for non-small cell lung cancer, renal cell carcinoma, and glioblastoma. Since then, many such drugs have followed suit and received approval, both monoclonal antibodies to VEGF and treatments which target other pathways. These treatments have impacted numerous other malignancies, including pancreatic neuroendocrine tumors, medullary thyroid cancer, and hepatocellular carcinoma [3].

The non-cytotoxic nature of anti-angiogenic drugs provides a therapeutic advantage over many traditional chemotherapy agents, which generally target disruption of cellular mitosis in both healthy and cancerous cells. However, a relative disadvantage of anti-angiogenic drug use is difficulty in determining the most effective dose and treatment duration since optimal regimens are difficult to establish [1]. Unfortunately, the breadth of research dedicated to antiangiogenic therapy is challenged by the emergence of drug resistance, specifically with the use of monotherapies. However, recent studies suggest that anti-angiogenic therapy may actually normalize intra-tumoral blood vessels, thus making neo-vasculature more sensitive to cytotoxic chemotherapy [4].

Since 1980, imaging has played an important role in the evaluation of tumor angiogenesis in preclinical models [5]. However, there is a wide gap in detection and quantification of angiogenesis between various clinical imaging and microscopic modalities. For example, conventional US, CT, and MRI can provide detailed information about gross tumor size and morphology, in addition to the vascularity pattern of large vessels. However, details about the microvascular environment are limited with these studies. More advanced dynamic imaging techniques, such as CT perfusion, are able to measure blood flow, blood volume, and vascular permeability. However, they are unable to provide specific information about alterations in vasculature and do not directly correlate with changes in vascular permeability, vascular density, or tumoral neo-angiogenesis [6]. Fluorescence-based imaging and electron microscopy are other options which may yield more detailed information about the structure, density, and permeability of the blood vessels. An example is seen in retinal and choroidal imaging, where fluorescent dye exposes leaky capillaries and detects ocular angiogenesis, visible externally through the eye. However, fluorescence imaging has its own limitations; most importantly, it is unable to provide accurate quantitative information [7].

Current research is focused on determining imaging regimens for follow-up of anti-angiogenic therapy. Efforts are also dedicated to improving both imaging diagnostic accuracy and anti-angiogenic interventional therapies [2]. Specifically, the discovery of a validated imaging biomarker will not only facilitate more accurate follow-up of anti-angiogenic therapy but also aid in determining correct therapeutic dose and treatment duration through improved evaluation of biological processes [6,8]. For example, dynamic contrast-enhanced (DCE) MRI with gadolinium-diethylenetriamine-penta-acetic acid (Gd-DPTA) labeled albumin has been found to be a reliable marker in detecting early response to bevacizumab in breast cancer animal models, with measurable reduction of vascular permeability at different doses [9].

## 2. Molecular Basis of Angiogenesis

**Figure 1:**
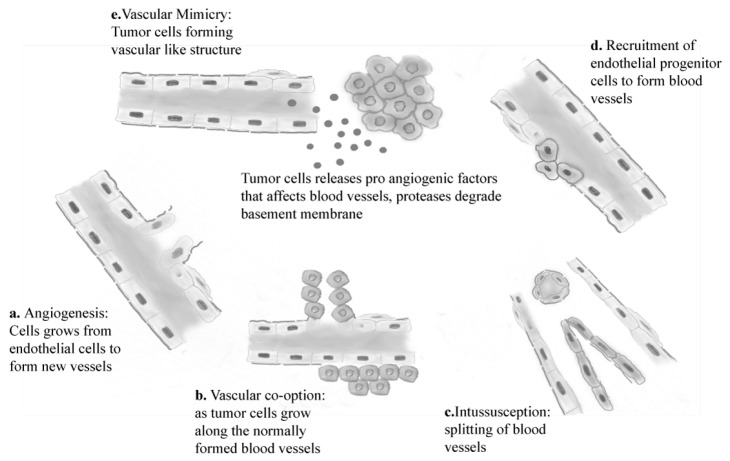
Graphic representation of various routes leading to new blood vessel formation within a tumor. (**a**) Angiogenesis, (**b**) vascular cooption, (**c**) intussusception, (**d**) endothelial progenitor recruitment, (**e**) vascular mimicry.

Under normal physiological conditions, angiogenic factors are strictly controlled by cellular machinery [10]. However, this meticulous management becomes unregulated and disorganized in neoplastic processes. During rapid tumor growth, neoplastic cells must be within millimeters of feeding vessels, as oxygen diffusion is limited to approximately 250 μm [11]. To maintain an adequate blood supply, tumor cells direct host cellular machinery via hypoxia-induced angiogenesis. New blood vessels form when cellular production of angiogenic factors outweighs the manufacture of anti-angiogenic factors (Figure 1) [10,12].

Tissue hypoxia stimulates the production of VEGF and fibroblast growth factor (FGF). In response, endothelial cells produce proteases that dissolve cellular basement membranes. This process propagates the formation of vascular buds, which eventually evolve into functional new blood vessels. Subsequent recruitment of accessory cells produce the necessary supporting extracellular matrix for newly formed vessels [2,13].

Although de novo formation of blood vessels, known as neovascularization, commonly occurs in malignancies, additional angiogenic pathways can also result in new vessel formation (Figure 1). These are the same processes that occur in physiologic blood vessel formation and include intussusception, when a single vessel splits into two vessels without cellular mitosis, and vascular mimicry, whereby tubular vessel-like structures form [14]. Tumor stem cells can also differentiate directly into endothelial cells, which begins new vessel formation [8].

Angiogenesis is governed by multiple angiogenic factors that may represent targets for novel molecular-based therapies. Some of these angiogenic elements include hypoxia-induced factors, growth factors, VEGF, platelet-derived growth factors, FGF2, matrix metalloproteinases, alpha v beta 3 integrin, and E-selectin [14]. Among these, VEGF is considered the most important angiogenic factor in the formation of new blood vessels [15]. Unlike other growth factors such as fibroblast growth factor (FGF) that stimulates mitosis in multiple cell lineages, VEGF is specific for stimulating vascular endothelial cells and thus angiogenesis [16].

**Figure 2:**
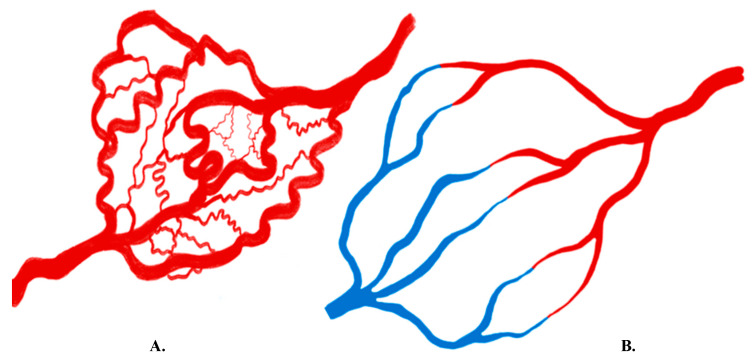
(**A**) Schematic representation of tumor vasculature showing disorganized architecture of the neo-angiogenesis with arteriovenous shunting. (**B**) Schematic representation of normal vasculature showing well organized arteriovenous capillary formation, red represents oxygenated blood and blue represents deoxygenated blood. (adapted from: Imaging Angiogenesis: Applications and Potential for Drug Development. Janet C. Miller, et al. Journal of the National Cancer Institute, 2005.

Many architectural and functional differences exist between vessels formed under physiologic and neoplastic conditions. For example, tumoral angiogenesis results in the rapid overproduction of angiogenic factors, which makes newly formed blood vessels often disorganized and highly permeable to macromolecules [17,18,19]. Vascular endothelial cadherin is crucial for maintaining cell–cell junctions, thus maintaining vascular integrity. VEGF activates the intracellular signaling pathway that downregulates VE-cadherin molecules, a major contributing factor in increasing vascular permeability [20]. Compared to orderly physiologic vessel formation, tumor vessels may haphazardly course throughout surrounding tissues and branch in atypical patterns [1]. Tumoral vessels, which are often not uniformly distributed across tumor tissue, demonstrate an increased number of blood vessels per unit area, known as microvascular density (MVD). Additionally, disorganized blood vessel formation can lead to intra-tumoral arteriovenous (AV) shunting. Finally, tumoral angiogenesis can result in blood flow fluxes out of the vascular compartment into the interstitial compartment due to increased oncotic pressure. This oncotic pressure increase is caused by leakage of plasma proteins into the interstitium through weak tumor vessel walls (Figure 2) [17,18,19].

Knowledge of the key differences between normal and abnormal vessels assists clinicians with the diagnosis, treatment, and prognosis of various neoplastic processes. The various structural and functional differences between physiologic and tumoral vessels are appreciated with various molecular and non-molecular imaging methods, including cross-sectional imaging and digital subtraction angiography (DSA) (Figure 2).

## 3. Overview of Imaging Angiogenesis

There are two main methods for imaging angiogenesis in tumoral tissue, including structural and functional assessments. Structural or anatomic evaluations involve molecular and non-molecular imaging, which include CT, MRI, contrast-enhanced ultrasound (CEUS), whole body optic imaging (after tumor labeling with green fluorescence), and positron emission tomography (PET) scan [7]. The molecular method relies on intravenous contrast, which contains nanoparticles, to bind specifically to ligands upregulated on the surfaces of neo-angiogenic vessels [21,22]. Conjugating the nanoparticles to surface ligands on actively proliferating endothelial cells bridges morphological imaging techniques and evaluation of angiogenesis at the molecular level [23].

The second method for evaluating angiogenesis focuses on cellular function and involves various perfusion parameters. These factors include blood flow, mean transient time, regional blood volume and assessment of vascular permeability. Vascular permeability is assessed with dynamic contrast-enhanced MRI or CT [21]. This parameter is significantly valuable as it correlates with vascular integrity and pathological grade in some tumors [24,25]. For example, the assessment of vascular permeability using MRI techniques could differentiate various grades of gliomas with high accuracy, particularly when combined with perfusion studies [26].

## 4. Invasive and Non-Invasive Imaging Methods

**Figure 3:**
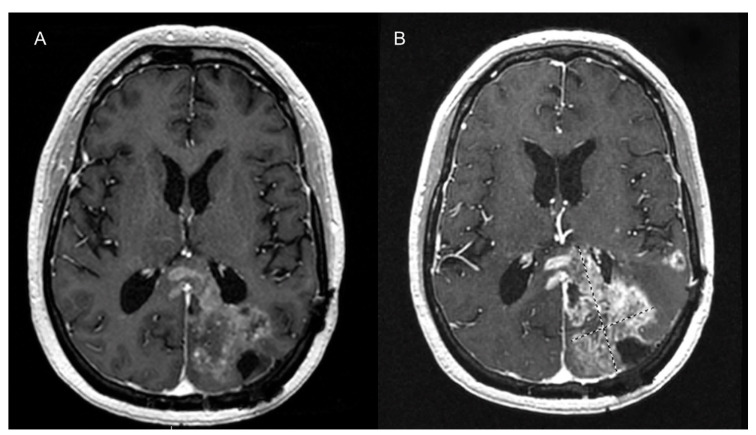
T1-weighted contrast-enhanced axial image of the brain with a left occipital glioblastoma. (**A**) This image shows enhancing local tumoral recurrence, prior to starting anti-angiogenic therapy. (**B**) Nine months after initiation of anti-angiogenic therapy shows marked reduction of the enhancing component and associated vasogenic edema.

**Figure 4:**
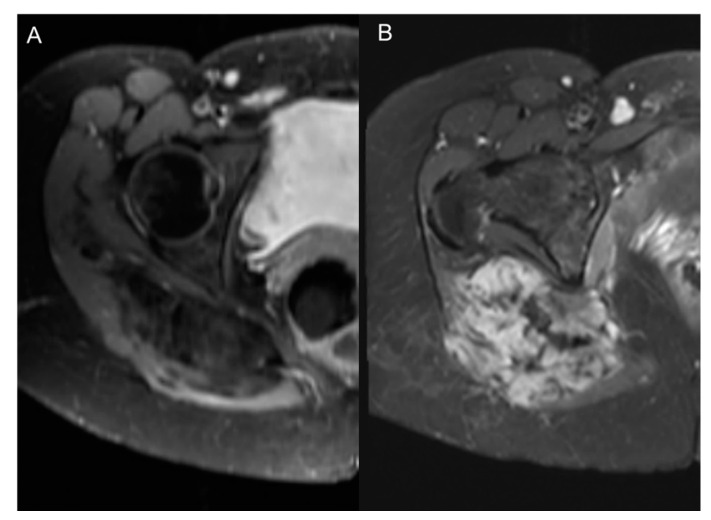
Axial T1 fat suppression contrast-enhanced MRI images of the pelvis in a female patient with fibro-desmoid tumor of the gluteal region. (**A**) Post- and (**B**) pre-treatment images after initiation of anti-angiogenic therapy.

**Figure 5:**
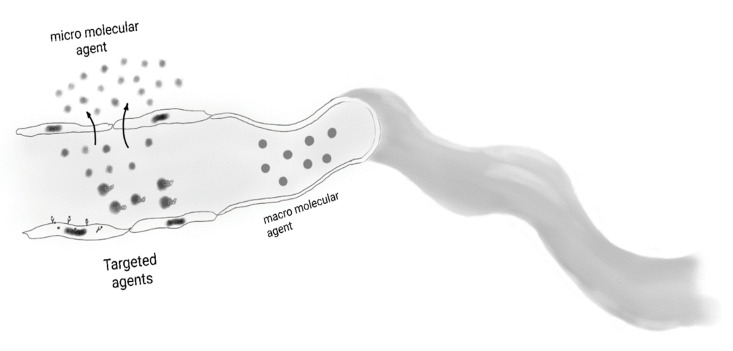
Graphic illustrates various contrast medias used in contrast-enhanced MRI, such as ultrasmall superparamagnetic iron oxide (USPIO). The small molecular agents leak through the vessel walls. Macromolecules remain in the vascular compartment. Similarly, ligand-bound contrast material remains in the vascular compartment. Targeted agents are bound to surface target molecules.

Non-invasive imaging methods primarily involve MRI and CT. MRI has various types of contrast agents that can be used. Low-molecular-weight MRI contrast agents, such as the now discontinued gadofosveset trisodium, extravasates at sites of angiogenesis, but also can leak from normal blood vessels. The most commercially used MRI contrast agents are macromolecular, such as Gd-DTPA, and are retained within normal vasculature but leak from vessels formed through a pathologic angiogenesis process, typical of tumors (Figure 3 and Figure 4). Ultrasmall superparamagnetic iron oxides (USPIOs) are high-contrast MRI contrast agents which are very small and are useful in assessment of the blood pool. Currently, they are more frequently utilized in research settings but are helpful in evaluating vessel characteristics, such as size or density [27] (Figure 5).

However, CT imaging with iodinated contrast is utilized as well, but it introduces ionizing radiation. Ultimately, the decision of CT or MRI is usually made on a case-by-case basis and is often dependent on the region being imaged and institution preference.

**Figure 6:**
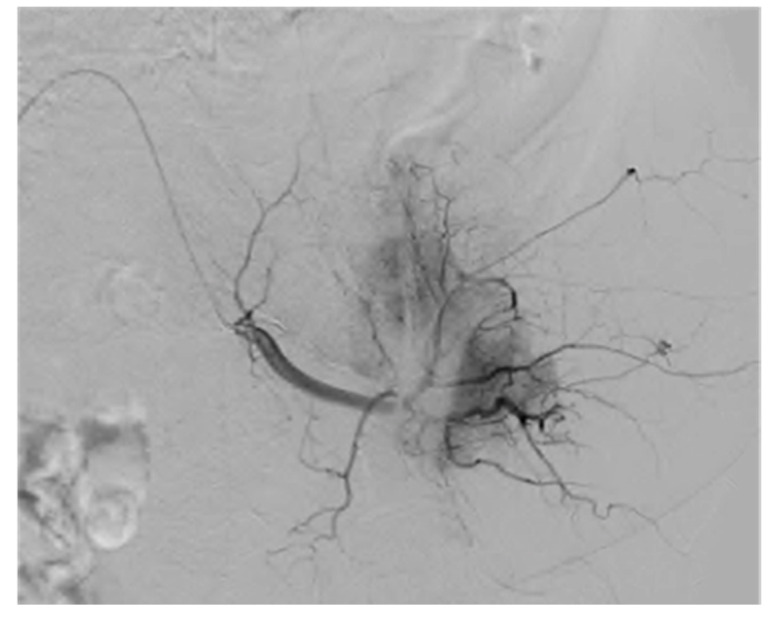
Digital subtraction angiography in a case of a metastatic renal carcinoma lesion in the left acetabulum. During the embolization procedure, there is significant tumoral vascular blush, denoting extensive intratumoral vessel formation.

Catheter angiography/DSA is a more invasive method where contrast is injected directly in the artery proximal to the tumor. This can be achieved using iodinated contrast agents or carbon dioxide (CO2). CO2 has an advantage as it is not nephrotoxic and does not have the risk of allergic reactions like iodinated contrasts [28]. Regardless, evaluation is typically limited to vessels larger than 250 µm in typical angiography equipment, which makes microvascular assessment impossible [28]. Much like CT, these methods also carry the risk of ionizing radiation [29] (Figure 6).

Even more invasive imaging methods quantify tumor microvascular density (MVD), which is an angiogenesis biomarker calculated from pathologic specimens, usually obtained after biopsy. Theoretically, acquisition of multiple biopsies throughout the course of the disease would provide information regarding response to anti-angiogenic therapy. However, MVD values are not entirely reliable because most tumors are not homogenously vascular [30]. For example, most areas of active angiogenesis are located at a tumor’s periphery compared to the less vascularized necrotic core [31].

When comparing techniques, imaging biomarkers may be advantageous over molecular biomarkers as the former is less invasive and has the added benefit of providing global information about tumor heterogeneity [8,32]. Although imaging biomarkers, such as perfusion studies, mean transient time, relative blood volume, and contrast transfer coefficient, are promising parameters for anti-angiogenic treatment response, these biomarkers still require validation in the clinical setting for correlation of imaging findings and treatment response [33].

## 5. Choosing the Best Imaging Modality for Evaluating Angiogenesis

In various clinical trials evaluating angiogenesis, the imaging modality of choice (MRI versus CT) is based on the availability [34]. The volume transfer coefficient (K_trans_) values, which combine both blood flow and vascular permeability surface area product, were comparable between these two modalities [33]. However, many studies that used diagnostic imaging for the evaluation of angiogenesis utilized MRI [35]. Additionally, CT and MRI offer comparable results regarding spatial resolution, although MRI can give information about water diffusion, tissue oxygenation and metabolism, microvascular permeability, and blood flow. PET imaging also appears to be a useful and highly accurate method for the assessment of changes in tumor vascularity [36]. However, its use is somewhat limited due to the lack of widespread availability and poor spatial resolution [17]. Perfusion CT imaging and CEUS are other options which have been utilized for evaluating angiogenesis [37]. Perfusion CT can offer quantitative data about blood flow similar to MRI; however, it carries the risk of exposure to ionizing radiation and also has a poorer signal to noise ratio [19]. With the recent development and advancement of CEUS, it is now possible to evaluate the vascularity of malignant tumors and has shown promise in clinical applications [38]. However, it can be limited in its ability to provide full 3D morphologic details of tumors and presents challenges in anatomically inaccessible regions, such as the retroperitoneum and brain [19].

## 6. Parameters Derived from Imaging Data

### 6.1. Steady-State Imaging

By comparing image intensity before and after the administration of contrast, blood volume at the region of interest can be calculated, which can be used for follow-up of drug therapy. However, a disadvantage of contrast-enhanced MRI and CT is that contrast may leak through the endothelial barrier in disorganized tumoral neo-vasculature, thus yielding inaccurate measures of blood volume. In comparison, microbubble contrast used in CEUS remains within the intravascular compartment and does not leak through vessel walls like traditional contrast agents, and thus is more accurate for measuring blood volume [1].

### 6.2. Dynamic Imaging

A large amount of useful information can be derived from dynamic studies, such as blood volume and blood flow, which may be particularly useful in assessing treatment response and tumor vascular dynamics. Provided that contrast remains within the intravascular compartment during ultrafast imaging techniques, the amount of contrast in each voxel of the image can be tracked and plotted against time such that the area under the curve will correspond to the blood volume [1]. This technique has previously been validated to provide important clinical information. For example, Zhang et al. demonstrated the prognostic value of regional cerebral blood volume for evaluating treatment response in cases of glioblastoma multiforme [39].

Flow can be calculated in similar ways, and although there are several methods, it generally is a measure of how fast the tracer (contrast) diffuses through a tissue [40]. It also yields a variety of useful parameters (Table 1).

## 7. MR Imaging of Angiogenesis

### 7.1. Unenhanced MRI Imaging of Angiogenesis

**Figure 7:**
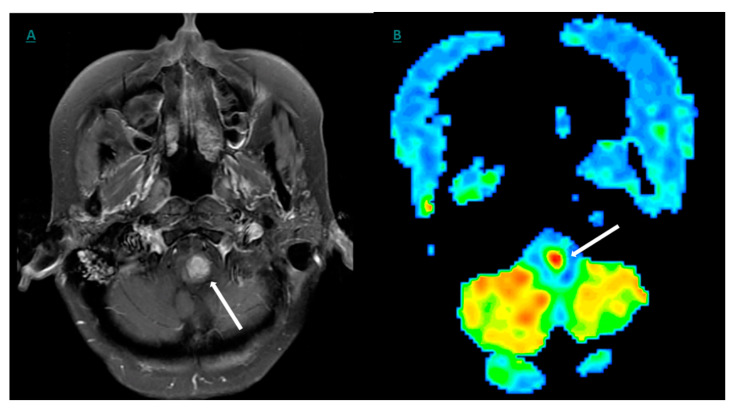
(**A**) DCE MRI axial T1-weighted image showing an enhancing cervicomedullary junction lesion (arrow). (**B**) The corresponding Arterial Spin Labeling (ASL) showing a relatively increased perfusion in the cervicomedullary junction (arrow).

Two non-contrast MRI techniques utilized in the evaluation of angiogenesis include arterial spine labeling (ASL) and blood oxygenation level-dependent (BOLD) imaging. ASL is a non-invasive, non-contrast method in which blood is magnetically labeled as it passes towards the organ of interest using RF pulse. The labelled protons interact with the tissue of interest, generating information use to indirectly calculate blood hemodynamics [38]. Using the same principle, ASL is used to generate blood flow maps, such as cerebral blood flow. Similarly, this technique could be used to indirectly evaluate the state of angiogenesis in a tumor (Figure 7) [39].

The applicability of ASL to extracranial tumors is limited due to transient time artifact, such as those produced by patient movement or respiratory motion. Nevertheless, this technique can be extrapolated to assess vascular changes in various extracranial tumors, like renal and ovarian cancers [40,41]. This method is particularly useful in renal cancer patients with renal failure, as no contrast is required. When compared to conventional T1-weighted contrast-enhanced MRI, ASL comparably detects renal cell carcinoma relapses after radiofrequency ablation [42].

The BOLD imaging technique is based on the fact that oxy and de-oxy hemoglobin exhibit different magnetic characteristics. This technique was initially developed for functional brain imaging, yet due to arteriovenous differences in blood oxygenation, indirect calculation of blood flow can be measured. However, it should be noted that this technique is limited by poor spatial resolution and susceptibility artifacts [43].

### 7.2. Contrast-Enhanced MR Imaging of Angiogenesis

Leakage of injected contrast from tumor angiogenic vasculature provides the basis for contrast-enhanced MRI studies. Furthermore, the high spatial resolution of contrast-enhanced MR imaging detects vessels as small as 30–100 micrometers [44]. DCE MRI is based on subjective enhancement curves, which identify angiogenesis in tumoral tissue [45]. It can be performed using either low-molecular-weight (LMW) or high-molecular-weight (HMW) contrast agents [46]. A frequently utilized LMW contrast agent for detection of vascular leakage is albumin-Gd-DTPA, which creates signal enhancement as it leaks into interstitial planes (Figure 5 and Figure 6) [9].

DCE MRI is performed with a T1-weighted technique to determine perfusion parameters and contrast material leakage. On the other hand, a T2* imaging technique is utilized to calculate cerebral blood flow and is optimized for the assessment of malignancy [47]. In a clinical trial investigating invasive ductal carcinoma, this T2* imaging technique exhibited a positive correlation between perfusion parameters and microvascular density, especially in larger tumors [45]. Thus, perfusion and DCE MRI techniques can be used to follow-up anti-angiogenic therapy response. Another study, which used colon cancer mouse models treated with bevacizumab, utilized DCE-MRI to assess the maturity and normalization of vessels, while BOLD-MRI was used to evaluate for tumor ischemia and metastatic potential. Using these methods, they concluded that low-dose intermittent treatment with bevacizumab may supplement the use of chemotherapy.

The parameters derived from DCE MRI include the volume of extravascular extracellular space per unit volume, volume of blood plasma per unit volume of tissue, blood vessel permeability, vascular surface area, and interstitial pressure [48,49]. These parameters may be applied to pharmacokinetic studies to evaluate for drug response, tumor recurrence, and progression [48].

### 7.3. Dynamic Susceptibility Contrast (DSC) Perfusion MRI

Dynamic susceptibility contrast (DSC) perfusion MRI, also termed bolus-tracking MRI, depends on the first pass metabolism of the contrast agent in the tissue. This technique involves injecting gadolinium chelate into the vascular compartment and then rapidly acquiring images through the region of interest. As contrast passes through the tissue of interest, the perivascular magnetic field is distorted which leads to T2 de-phasing and loss of signal intensity. When the measured signal intensity is plotted against time, hemodynamic parameters like blood volume and flow are calculated [50].

### 7.4. Dynamic Contrast-Enhanced Perfusion MRI

**Figure 8:**
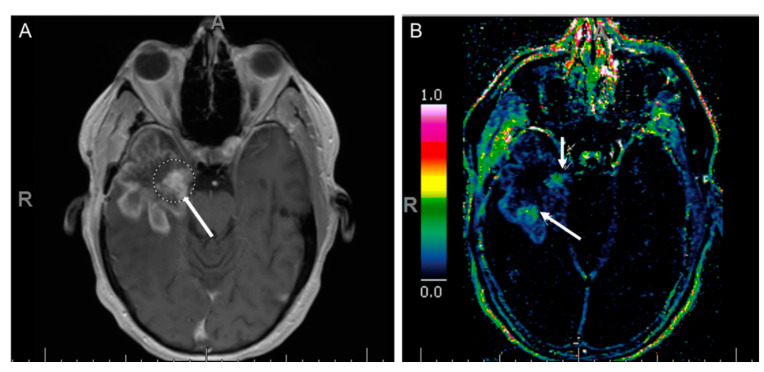
A patient with temporal glioblastoma underwent surgical resection followed by radiotherapy. (**A**) Axial T1 post-contrast image of the brain shows nodular enhancement. (**B**) This corresponds to K_trans_ map with increased capillary permeability (arrows). These regions show increased relative cerebral blood flow (rCBF) (not shown), which likely represents recurrence of disease.

**Figure 9:**
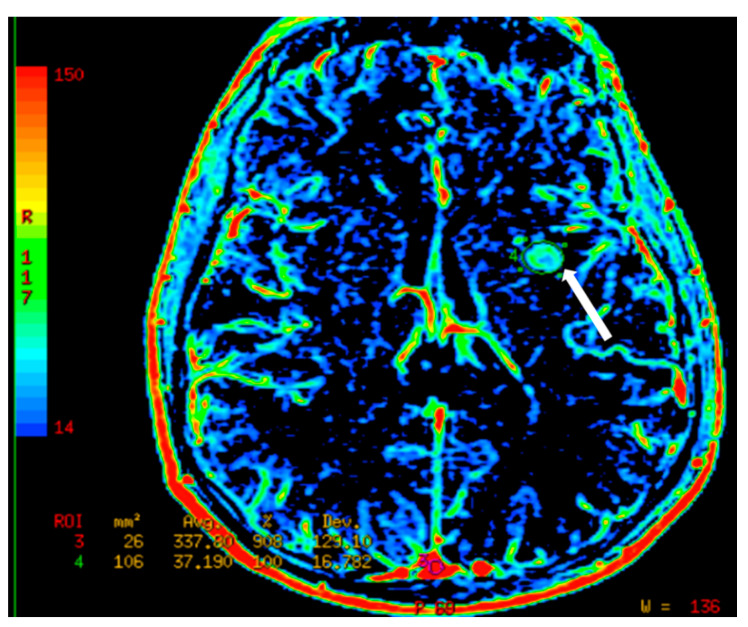
Axial dynamic contrast-enhanced (DCE) MRI of the brain with a relative cerebral cerebral blood volume (rCBV) map, which shows relatively increased flow dynamics in the left basal ganglia lesion (arrow).

With DCE perfusion MRI, T1 shortening is plotted against time. The contrast material diffuses into the extravascular extracellular space in a process dependent on vascular permeability, surface area, and tissue perfusion. By analyzing a specific region of interest, parameters like K_trans_ and V_p_ can be calculated from dynamic contrast-enhanced T1 MRI images (Figure 8) [51]. V_p_ is a measurement of the fractional plasma volume and is similar to blood volume. K_trans_ reflects the porosity at the capillary bed and is calculated by the rate that the MRI contrast material accumulates in the interstitial fluid compartment (Figure 8 and Figure 9).

When a contrast material is injected during dynamic studies, it diffuses through the capillary bed into the extravascular compartment at a rate dependent upon the permeability of the capillary bed and the concentration difference between the plasma and that volume of distribution [52].

## 8. CT Imaging of Angiogenesis

### 8.1. Multi-Slice Computed Tomography (MSCT) Perfusion Imaging Technique

CT imaging is often considered the gold standard for diagnosis, staging, and follow-up of tumors due to its superior spatial resolution with the contrast agents. From contrast-enhanced CT imaging, parameters like vessel size, relative blood volume and three-dimensional anatomy can be derived. However, these parameters represent macroscopic vasculature and fail to provide information at the micro capillary level [53]. Previously, a study determined a positive correlation between malignant breast mass perfusion parameters and increased expression of angiogenic factors where there was over-expression of VEGF and matrix metalloproteinase-2 (MMP-2) [54].

### 8.2. Clinical Applications of CT Perfusion

**Figure 10:**
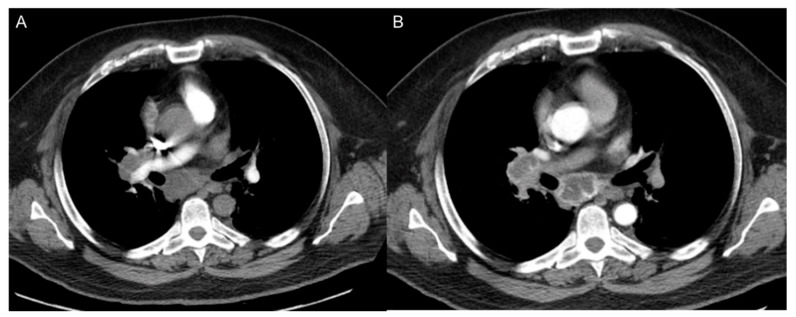
CT perfusion of a right hilar lesion was performed following administration of IV contrast. Displayed are selected sequential acquisitions (**A**,**B**) collected for measurement of perfusion parameters.

There are a few significant clinical applications of CT perfusion imaging. Lesion characterization can be achieved by analyzing perfusion values [55]. Previously, it has been shown that differences in perfusion was able to differentiate renal cell carcinoma from oncocytoma. These perfusion differences also extended to differentiate malignant renal tumors from normal renal cortical tissue [56]. Detection of tumoral angiogenesis with CT perfusion imaging is useful in staging tumors, particularly when evaluating perirectal tumors [55]. Additionally, it has been demonstrated to help with detecting nodal metastasis from squamous cell carcinoma and breast cancer (Figure 10) [57,58]. CT perfusion imaging may also predict which patients will respond better to chemo and radiotherapy. This is evidenced by the association between higher blood flow values in tumors which had a more robust treatment response [21]. Finally, some evidence suggests that this technique may also be utilized to detect relapse and evolvement of drug resistance [59].

## 9. Ultrasound Imaging of Angiogenesis

The development of high-frequency ultrasound machines permits real-time imaging of angiogenesis with excellent imaging resolution [60]. A combination of power doppler and CEUS quantifies blood flow and characterizes tumoral vascular structures. In experimental models, this combined technique can conduct evaluations for treatment responses to anti-angiogenic drugs [61].

Contrast-enhanced ultrasound uses microbubbles, which are gas filled spheres measuring 1–3 μm surrounded with a single lipid layer. This gas–fluid interface provides an excellent medium for backscattering of ultrasound waves. This happens when the waves interact with injected microbubbles producing nonlinear backscattering. This interaction produces real-time, contrast-specific images that can be viewed side by side with subtracted images. Even at small concentrations, contrast material can be detected as well as very sluggish flow within the tumor microvasculature [62]. By plotting enhancement versus time, maximal enhancement peak and relative blood volume are calculated. Perfusion is interpreted by the upslope of this curve [63]. The potential utility of microbubble CEUS in evaluating angiogenesis is evident in animal studies. In a study by Sirsi et al., the use of microbubble ultrasound revealed a decreased relative blood volume in tumors after anti-angiogenic therapy. Additionally, these findings correlated with the microvascular density on histological specimens [64].

There have been advances in developing target specific contrast agents. Lipid shells of microbubbles are modified to bind specifically to a transmembrane receptor. The same concept does not apply to soluble proteins (non-membrane-bound proteins) such as matrix metalloproteinase. However, these soluble proteins have to be present in sufficient concentration at the vascular compartment in order to bind to protein specific contrast agents [62].

A similar sonographic technique for evaluating angiogenesis is molecular ultrasound imaging, which uses antibodies bound to the microbubble contrast rather than just altering the lipid shells. These antibodies bind vascular surface receptors, which are over-expressed by many tumors, including vascular endothelial growth factor receptor (VEGFR). When combined with subtracted images, regional areas of target overexpression are revealed. In one study, this method showed a significant reduction of contrast binding after antiangiogenic therapy. The suggestion of this finding is a resultant decreased level of VEGFR expression after therapy and, thus, decreased angiogenic activity in the tumor tissue [65].

## 10. Positron Emission Tomography (PET) and Molecular Imaging

PET imaging is advantageous for patients as the tracers are generally less toxic compared to MRI and CT contrast agents [66]. Additionally, the radiotracers can be conjugated to various substances or ligands, allowing for the ability to target specific receptors or cells. This is often referred to as molecular imaging. For example, radiolabeled VEGF or antiangiogenesis agents can be used in PET to provide evidence that the treatment is targeting the tumor tissue [15]. This imaging technique can also demonstrate the heterogeneity of targeted molecules distributed within the tumor tissue [67]. There are a number of other targets and radiopharmaceuticals that have been studied and utilized, further discussed below (Table 2).

## 11. 18F-FDG- (Fluorine 18 Fluoro-Deoxy-Glucose)-Based Imaging

18F-FDG-based PET is the most frequently used radiotracer in the clinical setting. Its use in evaluating malignant lesions is dependent on the increased glucose metabolism in malignant tumor cells. However, its role in predicting tumor response to anti-angiogenic therapy is promising but not yet fully validated. Previously, a study revealed a strong association between tumor angiogenesis-related gene expression and 18F-FDG uptake [74]. There are several others which support this method, including a more recent study of renal cell carcinoma metastasis by Hwang et al. concluding that metabolic tumor volume and lesion glycolysis could provide prognostic information in patients treated with anti-VEGF therapy [15]. Investigations are ongoing to fully validate 18F-FDG PET in clinical use for evaluating tumor response to anti-angiogenic therapy.

### 11.1. VEGF and VEGFR Targeting

The targeting of VEGF or its tyrosine kinase receptor (VEGFR) via radiopharmaceuticals in PET or single-photon emission computerized tomography (SPECT) imaging is an exciting prospect. This technique may play an important role in the evaluation of anti-angiogenic treatment response since VEGFR-2 is upregulated in many neoplasms [75]. Preclinical studies reveal the possibility of radiopharmaceutical binding to VEGFR. This explains its role in imaging heterogeneity of tumor vasculature in animal breast cancer models and also its role in following up response to antiangiogenic drugs. However, these radiopharmaceutical agents must be refined for better understanding of their in vivo pharmacokinetics and to obtain a more potent affinity for VEGF [15].

### 11.2. Targeting Extracellular Matrix (ECM)

Fibronectin is an extracellular matrix protein that is expressed in normal tissues. Through alternative mRNA splicing, it can exist as an isoform that expresses an extra domain-B (ED-B). This isoform is found predominantly within fetal and neoplastic adult tissues and is often absent from healthy adult tissues. Malignant cells grow over a background of extracellular matrix proteins that expresses ED-B fibronectin. Thus, this isoform may be a potential target for molecular imaging evaluation of anti-angiogenic treatment response [76].

Other targets for radionuclide-based imaging include the integrins, predominantly alpha v beta 3, which are adhesive glycoproteins for extracellular matrix molecules. Both the alpha and beta subunits play an important role during angiogenesis, as they are upregulated in tumor metastasis to activate matrix metalloproteinase (MMP) that breaks down extracellular matrix [77]. Alpha v beta 3 overexpression is observed to be related to tumor aggressiveness and metastatic potentials. For example, Van Belle et al. discovered that these proteins are integral in advancing melanoma transformation beyond radial superficial growth to deep vertical growth that denotes invasive pre-metastatic behavior [78].

### 11.3. Other Potential Targets

Matrix metalloproteinases (MMP) are a group of zinc-dependent proteolytic enzymes with both pro- and anti-angiogenic activities. They are released during angiogenesis to degrade cellular basement membranes and permit branching of newly formed blood vessels. Previously, MMP-2 has been successfully targeted and imaged in mouse models by attaching an MMP-2 inhibitor to Galium-68, a radioisotope currently used in clinical practice [79]. These proteinases may serve as molecular targets for diagnostic and therapeutic interventions in the future [80].

Aminopeptidase N (APN) is a membrane-bound enzyme that is responsible for amino acid cleavage and has previously been shown to be involved in tumor angiogenesis [81]. Importantly, APN is absent within normal vasculature but has high expression within tumoral vessels undergoing angiogenesis [82]. This feature makes it a great target for evaluating angiogenesis activity within a tumor, and potentially for evaluating response to anti-angiogenesis agents. Studies have previously conjugated an APN-targeting agent to copper-68, a positron emitter used it PET, and technetium-99, an agent used in SPECT. Imaging was then performed and their utility demonstrated within mouse models [83,84].

## 12. Emerging Imaging Techniques for Angiogenesis

### 12.1. Ultrasound

Advances in ultrasound technology have introduced ultrafast techniques that deliver very high resolution and sensitivity. This allows for detailed characterization of the microvascular environment as well as quantification of blood flow without contrast [85]. Additionally, this technique has been shown to detect sluggish blood flow, as evidenced in rat models’ microvasculature [11,86].

Ultrasound with contrast pulse sequences is a real-time perfusion imaging technique with a high specificity and spatial resolution. Multi-pulse contrast imaging suppresses linear back scattering from tissue (noise) while detecting a non-linear signal from the contrast agent (microbubbles). This allows for better assessment of small microvasculature where there is sluggish blood flow and a small concentration of microbubbles. In experimental studies, this technique produces results that are comparable to dynamic contrast-enhanced MRI in terms of perfusion parameters. In addition, contrast-enhanced ultrasound provides real-time imaging and detailed perfusion information during a single cardiac cycle [85].

### 12.2. Photoacoustic Imaging (PI)

Photoacoustic imaging is a relatively new advancement demonstrating promising results in clinical imaging. This technique utilizes optical waves (light) to vibrate a tissue, which then emits sonographic waves. A detector can then pick up the emitted wave and convert it to a usable image. There are several inherent advantages to this modality, including high-spatial and -temporal resolution and its ability to use endogenous substances as a contrast [87]. This includes hemoglobin, allowing the visualization of small vessels. Recently, a study performed using PI in prostate cancer patients found that imaging findings correlated well areas of microvascular density and vascular area, suggesting a possible use in assessing prostate cancer angiogenesis [88]. Another study used PI to identify blood vessel densities in breast tissue with high resolution. With this, they were able to identify breast cancer lesions accurately [89]. Despite the many promises of photoacoustic imaging, there is still much work to be done before widespread clinical adoption, let alone validating it for use in follow-up of anti-angiogenesis treatment.

### 12.3. X-ray Phase Contrast Imaging

This is a new method which has not yet made its way into clinical imaging but has had several successes in the research and pre-clinical settings. Much like conventional radiography, this method uses ionizing X-rays, but instead of only measuring the degree of beam attenuation, it also accounts for beam distortion and scatter caused by the imaged structure. This results in an image with much improved resolution. An early study performed using both a phantom and a rat kidney found that CO2 angiography resulted in a vessel resolution of around 60 µm with simulations predicting a resolution of around 20 µm in living tissue at reasonable radiation doses [90]. A follow-up study in a tumor model concluded that this method could be extended down to 15 µm to allow for tumor angiogenesis analysis [91]. However, much like the above, there is significant work that needs to be done before clinical adoption.

## 13. Assessing the Response to Anti-Angiogenic Therapy

By using anti-angiogenic drugs, it is postulated that tumor vascular density will decrease more significantly than tumor cells density [92]. If the tumor cells regresses faster than blood vessels, this in turn will increase the blood vessel index despite the decrease in tumor size; thus, the blood vessel index is rendered useless in evaluating anti-angiogenic response, giving a false impression of a stationary course [93].

Assessment of microvascular density in tissue biopsies is a straightforward, yet invasive, evaluation of therapy response [1]. However, the highest density of angiogenesis is found at the tumor periphery, while the core becomes de-vascularized and necrotic during tumor growth. Thus, obtaining comparable biopsies is challenging and could skew the observed response to treatment [31].

In summary, this review highlights various established and experimental methods for evaluating tumor response with anti-angiogenic therapy. While conventional imaging like CT, MRI, and US provide excellent anatomic characterization of tumor tissue, limited microvascular data are available. The addition of contrast agents to these conventional imaging modalities permits quantification of various perfusion parameters and provides insight on vascular function. Finally, molecular imaging utilizes a variety of targets to analyze anti-angiogenic therapy response on a microvascular level and provides a promising avenue for future advances. Regardless of the preferred imaging method, it is imperative to follow up anti-angiogenic treatment response. As these drugs pose many side effects to patients, it is essential to confirm a positive or negative therapeutic response to prevent the unnecessary continuation of these medications [21]. The use of anti-angiogenic factors also carries risk for thromboembolic events, mostly which result in myocardial infarction through switching the endothelial surface into a prothrombotic environment. Conversely, paradoxical bleeding is another complication that can be life threatening and difficult to manage, given the normal coagulation profile [94].

## Figures and Tables

**Table 1:** Summary of the measured and calculated hemodynamic parameters and their clinical significance.

Symbol	Measures	Significance
Cp (arterial concentration)	Concentration of tracer in arterial blood (input function in CT and MRI)	Arterial tracer concentration over time Shape of arterial input curve will correspond to the shape of arterial dynamics within the tissue itself
Ca (arterial concentration)	Concentration of tracer in arterial blood (input function in PET)	(as above)
F (flow)	Blood flow: represents the amount of blood passing per unit of time per tissue mass	Decreased under antiangiogenic therapy
P (permeability)	Capillary wall permeability surface area	Testing the integrity of the blood vessel mesh
PS (permeability-surface area product)	Capillary wall permeability surface area per unit mass of tissue (in practice, *P* and *PS* are not clearly separated)	Testing the integrity of the blood vessels mesh in relation to tissue volume
E (extraction fraction)	Extraction fraction of oxygen	Ratio of blood oxygen that a tissue takes up from blood flow
K_trans_ (volume transfer coefficient)	Volume transfer constant from plasma to the extravascular extracellular compartment	Predominantly dependent upon tissue perfusion and minorly dependent on permeability and surface area. Decreased with antiangiogenic therapy
K_ep_ (equilibrium product constant)	Rate constant between the extravascular extracellular compartment and the plasma (efflux of tracer back to the vascular space)	Predominantly dependent on vascular permeability and perfusion Variably changed under antiangiogenic therapy
Ki (net influx rate)	Unidirectional influx constant	
Vp (plasma blood volume)	Fractional blood plasma volume	
Va (arterial blood volume)	Fractional blood volume	
Ve (extravascular extracellular volume fraction)	Volume of extracellular extravascular per tissue unit volume	
IAUC (initial area under the curve)	Area under the curve in contrast concentration against time curve	This parameter is affected by perfusion and rBV Decreased under antiangiogenic therapy
rBV (relative blood volume)	Percentage of blood volume in relation to tissue volume	Amount of functional blood vessels within the tumor Changes according to the tumor vascularity Decreases with antiangiogenic therapy
Perfusion	Amount of blood flow within the capillary system (blood flow rate over organ mass)	Tumors will have higher perfusion rather than corresponding normal tissue due to increased microvascular density, larger arterial feeder. Decrease with anti-angiogenic therapy
MTT (Mean transient time)	Time a contrast agent has to pass through the tissue of interest from the arterial side to the venous draining side (blood volume over blood flow)	Correlates with tissue perfusion Decreases with anti-angiogenic therapy
Vascular permeability	Depends on various parameters like tracer properties, vascular surface area, leakiness and the extracellular extravascular distribution space	Tumors possess immature capillary mesh so are characterized by higher-than-normal vascular permeability

**Table 2:** Molecular targets in molecular imaging of angiogenesis.

Radiolabeled Substance	Example Radiotracer	Tracer Modality *	Significance
VEGF/VEGFR	[^64^Cu]VEGF_125-136_ [68]	PET	VEGF is a significant driver of tumor angiogenesis VEGFR-2 is upregulated in tumor angiogenesis Many approved anti-angiogenesis agents target VEGF/VEGFR
Bevacizumab	^89^Zr-bevacizumab [69]	PET	Bevacizumab targets VEGF-A Can evaluate biodistribution of treatment Assesses potential for response prior to treatment
Integrins (αvβ3)	^99m^Tc-3PRGD_2_ [70]	SPECT	Upregulated in angiogenesis and are involved with MMPs Overexpression seen in aggressive tumors
Matrix metalloproteinases (MMP)	^68^Ga-DOTA-TCTP-1 (MMP-2) [71]	PET	Involved in angiogenesis Degrades the basement membrane Permits branching
Fibronectin	^76^Br-L19-SIP (ED-B) [72]	PET	Absent in healthy adult tissue Found in neoplastic cells
Nitroimidazole	^18^F-FETNIM [73]	PET	Nitroimidazole is upregulated in hypoxic tissue Appears to play a role in angiogenesis

* The modality is dependent on the radiotracer used. Abbreviations: VEGF, vascular endothelial growth factor; VEGFR, vascular endothelial growth factor receptor; PET, positron emission tomography; SPECT, single-positron emission computer tomography; MMP, matrix metalloproteinases.
